# Kinetics of Phenotypic and Functional Changes in Mouse Models of Sponge Implants: Rational Selection to Optimize Protocols for Specific Biomolecules Screening Purposes

**DOI:** 10.3389/fbioe.2020.538203

**Published:** 2020-12-02

**Authors:** Mariana Ferreira Lanna, Lucilene Aparecida Resende, Rodrigo Dian de Oliveira Aguiar-Soares, Marina Barcelos de Miranda, Ludmila Zanandreis de Mendonça, Otoni Alves de Oliveira Melo Júnior, Reysla Maria da Silveira Mariano, Jaqueline Costa Leite, Patricia Silveira, Rodrigo Corrêa-Oliveira, Walderez Ornelas Dutra, Alexandre Barbosa Reis, Olindo Assis Martins-Filho, Sandra Aparecida Lima de Moura, Denise Silveira-Lemos, Rodolfo Cordeiro Giunchetti

**Affiliations:** ^1^Laboratório de Biologia das Interações Celulares, Departamento de Morfologia, Universidade Federal de Minas Gerais, Belo Horizonte, Brazil; ^2^Laboratório de Pesquisas Clínicas, Programa de Pós-Graduação de Ciências Farmacêuticas, Universidade Federal de Ouro Preto, Ouro Preto, Brazil; ^3^Laboratório de Biomateriais e Patologia Experimental, Instituto de Ciências Exatas e Biológicas, Universidade Federal de Ouro Preto, Ouro Preto, Brazil; ^4^Grupo de Pesquisa em Imunologia Celular e Molecular, Instituto de Pesquisa René Rachou, Fundação Oswaldo Cruz, Belo Horizonte, Brazil; ^5^Grupo Integrado de Pesquisas em Biomarcadores, Instituto de Pesquisa René Rachou, Fundação Oswaldo Cruz, Belo Horizonte, Brazil; ^6^Departamento de Medicina, Universidade José Rosário Vellano, Belo Horizonte, Brazil

**Keywords:** sponge implant model, biomolecules screening, dynamics of phenotypic and functional features, immunophenotyping, cytokines

## Abstract

The sponge implant has been applied as an important *in vivo* model for the study of inflammatory processes as it induces the migration, proliferation, and accumulation of inflammatory cells, angiogenesis, and extracellular matrix deposition in its trabeculae. The characterization of immune events in sponge implants would be useful in identifying the immunological events that could support the selection of an appropriate experimental model (mouse strain) and time post-implant analysis in optimized protocols for novel applications of this model such as in biomolecules screening. Here, the changes in histological/morphometric, immunophenotypic and functional features of infiltrating leukocytes (LEU) were assessed in sponge implants for Swiss, BALB/c, and C57BL/6 mice. A gradual increase of fibrovascular stroma and a progressive decrease in LEU infiltration, mainly composed of polymorphonuclear cells with progressive shift toward mononuclear cells at late time-points were observed over time. Usually, Swiss mice presented a more prominent immune response with late mixed pattern (pro-inflammatory/anti-inflammatory: IL-2/IFN-γ/IL-4/IL-10/IL-17) of cytokine production. While BALB/c mice showed an early activation of the innate response with a controlled cytokine profile (low inflammatory potential), C57BL/6 mice presented a typical early pro-inflammatory (IL-6/TNF/IFN-γ) response with persistent neutrophilic involvement. A rational selection of the ideal time-point/mouse-lineage would avoid bias or tendentious results. Criteria such as low number of increased biomarkers, no recruitment of cytotoxic response, minor cytokine production, and lower biomarker connectivity (described as biomarker signature analysis and network analysis) guided the choice of the best time-point for each model (Day5/Swiss; Day7/BALB/c; Day6/C57BL/6) with wide application for screening purposes, such as identification of therapeutic biomolecules, selection of antigens/adjuvants, and follow-up of innate and adaptive immune response to vaccines candidates.

## Introduction

The sponge implant has been applied as an important *in vivo* model for the study of inflammatory processes (Xavier et al., 2010; Pereira et al., 2012; Cassini-Vieira et al., 2014). In this sense, the sponge implant model, subcutaneously or intraperitoneally, is able to induce inflammatory angiogenesis, in which leukocytes (LEU) act as angiogenesis initiators (Mendes et al., 2009; Marques et al., 2011; Guedes-da-Silva et al., 2015). It is important to highlight that the subcutaneous implant sponge does not exhibit an intense inflammatory profile or a strong adherence to organs as described in intraperitoneal implants (Mendes et al., 2007).

After implantation in a subcutaneous compartment, the acellular and avascular synthetic sponge matrix induces the migration, proliferation and accumulation of inflammatory cells, angiogenesis, and extracellular matrix deposition in its trabeculae (Andrade et al., 1997; Moura et al., 2011c). The sponge implant model also allows the sequential study of the inflammatory infiltrate through histomorphometrical and biochemical analysis using myeloperoxidase (MPO) or *N*-acetyl-glucosaminidase (NAG) activities to indirectly determine neutrophils and macrophages, respectively (Bailey, 1988). Moreover, since tissue reaction to the sponge implant is circumscribed by a capsule of newly formed connective tissue, it is also possible to evaluate the cytokine profile in this microenvironment (Moura et al., 2011a). Although the sponge implant characterization has been broadly described in histomorphometric parameters (Xavier et al., 2010; Marques et al., 2011; Pereira et al., 2012; Cassini-Vieira et al., 2014; Guedes-da-Silva et al., 2015), the immune status in this compartment is poorly understood. To date, studies has focused on determining the cytokines (TNF-α, IL-6, IL-1β, TGF-β, IL-10, IL-17) and chemokines (CXCL1, CCL2, CCL3, CCL5) by the Enzyme Linked Immunosorbent Assay (ELISA) method (Barcelos et al., 2004, 2009; Belo et al., 2004; Agrawal et al., 2011; Alhaider et al., 2014b). In fact, the subcutaneous implantation of sponges has been used in several studies, since it is a model that induces an amplified inflammatory foreign body response that evolves into the formation of a highly vascularized granulation tissue in which various constituents can be analyzed (Andrade et al., 1997; Agrawal et al., 2011; Moura et al., 2011c; Alhaider et al., 2014b). Most of these studies are focused on the inflammatory angiogenesis model, since significant evidence indicates that angiogenesis and inflammation are key components to the maintenance of a variety of pathological conditions, whereas others seek to access the anti-angiogenic and/or anti-inflammatory outcomes of pharmacological compounds to prove its effects (Xavier et al., 2010; Agrawal et al., 2011; Moura et al., 2011a,c; Saraswati et al., 2011; Pereira et al., 2012; Alhaider et al., 2014a; Almeida et al., 2014; Cassini-Vieira et al., 2014, 2016; Michel et al., 2015). Regardless of the goal, the immunophenotypic characterization in the sponge implant was not employed in the analysis of LEU subsets.

Remarkably, the sponge implant can be used to analyze the immune response in a controlled microenvironment, which represents a preliminary step toward the development of an *in vivo* platform for trialing potential biomolecules. In fact, the immune response can be monitored by investigating the cell population and analyzing the compartmentalized microenvironment applied to trialing of anti-inflammatory or anti-angiogenic molecules (Xavier et al., 2010; Agrawal et al., 2011; Moura et al., 2011a; Saraswati et al., 2011; Alhaider et al., 2014a, b; Almeida et al., 2014; Cassini-Vieira et al., 2014, 2016; Michel et al., 2015).

The sponge implant model has been extensively studied over the last few decades (Andrade et al., 1987, 1997; Bailey, 1988; Barcelos et al., 2004, 2009; Belo et al., 2004; Mendes et al., 2007, 2009; Xavier et al., 2010; Agrawal et al., 2011; Marques et al., 2011; Moura et al., 2011a,b,c; Saraswati et al., 2011; Pereira et al., 2012, 2014; Alhaider et al., 2014a, b; Almeida et al., 2014; Cassini-Vieira et al., 2014, 2016; Guedes-da-Silva et al., 2015; Michel et al., 2015) and it has a broad practical application. However, little is known about the kinetics of the immunophenotypic and cytokine profile in the implant or about the changes in these parameters over time. The data regarding immune events into sponge implants would be useful to identify the LEU migration and their functional performance according to the cytokine microenvironment. Moreover, this information needs additional investigation based on the mice genetic background (Swiss, BALB/c and C57BL/6) considering the distinct time-points after sponge implantation. Also, in order to understand the complex immunological microenvironment that involves multiple events occurring simultaneously, the biomarker signature analysis was carried out based on a proposal published by Luiza-Silva et al. (2011). These data are required to support the choice of ideal time post-implant that present a minor inflammatory pattern. Importantly, the choice of the time post-implant presenting increased inflammatory biomarkers could induce interference in the sponge microenvironment and result in tendentious biomolecule screening results (therapeutic biomolecules and antigens/adjuvants). Herein, we studied the dynamics of phenotypic and functional changes in the sponge implant microenvironment that could ultimately support the selection of the best mouse lineage and time-point for screening assays as a relevant prerequisite to employing the sponge implant model for testing bioactive molecules. Our findings provided evidence to support the choice of the best model for biomolecule screening.

## Materials and Methods

### Animals

Male Swiss, BALB/c and C57BL/6 mice (8–10 weeks old; *n* = 20 mice/each time analyzed) were provided by the Centro de Ciência Animal (CCA) at Universidade Federal de Ouro Preto (UFOP), Brazil. The animals were kept in ventilated racks with food and water *ad libitum* throughout the study, with intermittent light/dark cycles every 12 h. The experimental design was approved by the Ethical Committee for Animal Studies (CEUA/UFOP, # 014/2011).

### Sponge Implants

**FIGURE 1:**
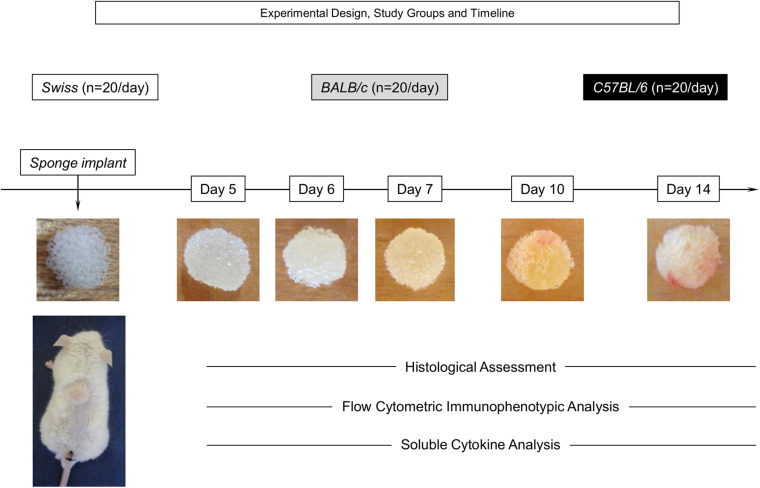
Experimental design, study groups, and timeline. The sponge disks were subcutaneously implanted and removed at Day5, Day6, Day7, Day10, and Day14 post-implant. A total of 100 Swiss, BALB/c, and C57BL/6 mice were used to assess, in duplicated experimental batches, the histological and morphometric parameters, flow cytometry immunophenotyping, and soluble cytokine measurements in independent sets of experiments at distinct time-points post-implant (20 animals/day).

Disk-shaped (4 mm × 8 mm) polyether-polyurethane sponges (Rei das Espumas, Belo Horizonte, Brazil) were soaked overnight in 70% v/v ethanol and boiled in distilled water for 15 min prior to implantation. Mice were anesthetized by intra-peritoneal injection of ketamine (150 mg kg^–1^) plus xylazine (10 mg kg^–1^) and the dorsal fur shaved and the skin wiped with 70% v/v ethanol. The sponge disks were subcutaneously implanted throughout a 1-cm long dorsal mid-line incision and the animals were monitored daily for discomfort/distress or any signs of opportunistic infection. Sponge implants were removed for histological/morphometric analysis, flow cytometry, immunophenotyping, and soluble cytokine measurements at Day5, Day6, Day7, Day10, and Day14 after implantation. The compendium of the experimental design, study groups, timeline, and illustrated images of sponge implants are provided in Figure 1.

### Histological and Morphometric Analysis

Sponges implants removed at Day5, Day6, Day7, Day10, and Day14 after implantation were weighted, fixed in 10% buffered formalin, pH 7.4, paraffin embedded, cut into 5 μm sections, mounted on slides, and stained by Hematoxylin/Eosin (HE) method. Quantification of inflammatory infiltrate (cell nuclei) and blood vessel counts were carried out by image acquisition of 25 random microscopic fields using a planapochromatic objective 40× under light microscopy (Leica DM5000B, Heerbrugg, Switzerland) using the Leica Application Suite software (version 2.4.0 R1, Leica Microsystems Ltd., Heerbrugg, Switzerland). Sponge weight was reported in micrograms. The results of the histological analysis were expressed as mean ± standard error (SE) of the total number of cell nuclei and blood vessels.

### Flow Cytometry Immunophenotyping

The LEU subsets harvested from sponge implants were quantified by flow cytometry at Day5, Day6, Day7, Day10, and Day14 after implantation. A panel of fluorescent monoclonal antibodies were employed including anti-CD45 (APC clone 30-F11/E071491630, FITC clone Sa230-F11/E003051630), anti-CD3 (Pe-Cy5 clone 145-2C11/E060661630), anti-CD8 (APC clone 53.6-7/E070561330), anti-CD49b (FITC clone HMa2/E001841630), anti-CD11c (Pe-Cy5 clone N418/E006121631), F4/80 (FITC clone BM8/E006121631) and LY6G (APC clone RB6-8C5/E001610630) from e-Bioscience (San Diego, CA, United States), and anti-CD4 (FITC clone RM4-5/714474^a^), Invitrogen (Carlsbad, CA, United States) (Supplementary Figure 1 describes the strategy for immunophenotyping analysis).

The sponges were removed from the implant site and incubated for 1 h in a solution containing trypsin, after which the sponge implants were gently squeezed at room temperature in 2 mL of RPMI and the sponge debris removed by differential centrifugation at 200×*g* for 5 min at 4°C. The supernatant was then centrifuged at 400×*g* for 8 min at 4°C to obtain the cell pellet. After resuspension of cell pellet the erythrocyte were lysed using 10 mL of ammonium chloride buffer. The LEU were washed once with 10 mL of RPMI at 400×*g* for 8 min at 4°C. The cell counts were determined with a Neubauer chamber and the final cell suspension adjusted to 1 × 10^5^ cells/mL. Aliquots of 100 μL of cell suspension (1 × 10^4^ cells per tube) were incubated in polypropylene tubes containing combinations of 20 μL of monoclonal antibody. Following incubation, stained cells were washed once with phosphate buffered saline (PBS) and resuspended in 250 μL of PBS. Non-specific binding was monitored by using fluorochrome-labeled isotypic matched reagents to provide valid negative controls. Autofluorescence was monitored by the use of a negative control in which the cell suspension was incubated in the absence of fluorochrome-labeled monoclonal antibodies, but in the presence of dilution and wash buffers. Flow cytometric measurements were performed on a FACScalibur^®^ instrument (Becton Dickinson, Mountain View, CA, United States). A total of 20,000 events were acquired for each sample. The CELLQuestPro software (Franklin Lakes, NJ, United States) was used for data acquisition and storage. FlowJo Software (Flow Cytometry Analysis Software Version 10.1, Tree Star, Inc., Ashland, OR, United States) was used for data analyses.

### Soluble Cytokine Measurements

The sponge implants were homogenized in 1 mL of RPMI using a tissue homogenizer (Homo mix). The homogenates were centrifuged at 3,000×*g* for 10 min at 4°C and supernatants stored at −80°C until processing. Soluble cytokine levels were measured by Cytometric Bead Array (BD Biosciences, San Jose, CA, United States), according to the manufacturer’s recommendations. The mouse inflammation kit was employed to measure the levels of soluble IL-2, IL-4, IL-6, IL-10, IL-17, IFN-γ, and TNF. FCAP software v.1.0.2 (BD Biosciences) was used for data analysis. The results were in picograms per milliliter (pg/mL) derived from the standard curves obtained for each cytokine.

### Statistical Analysis

#### Conventional Statistics

Intragroup comparative analysis among days post-implant and intergroup (all three mice lineages – Swiss, BALB/c, and C57BL/6) comparative analysis at a given day post-implant were performed by One-way ANOVA followed by Tukey’s multiple comparison test to compare all pairs of data. In all cases, significance was considered at *p* < 0.05. GraphPad Prism (version 5.03, San Diego, CA, United States) was used for statistical analysis and graphical arts.

#### Biomarker Signature Analysis

The biomarker signature analysis was carried out based on the pioneer proposal published by Luiza-Silva et al. (2011), initially proposed to highlight cytokine signatures of innate and adaptive immunity following 17DD yellow fever vaccination, which allows the identification of subtle differences that are usually not detectable by conventional statistical approaches, but are relevant to understanding the complex immunological microenvironments that involve multiple events. The biomarker signatures were determined by first taking into account the frequency of proportion of implants with biomarker levels above the global median cut-off defined for each biomarker. Briefly, to detect the global median cut-off, the data set was listed in numerical order from smallest to largest and then the median was calculated for each cell subset (CD45+, Ly+, F4/80+, CD11c+, CD3-CD49+, CD3+, CD3+CD4+, and CD3+D8+) as well as each soluble cytokine (IL-6, TNF, IL-2, IFN-γ, IL-4, IL-10, and IL-17) employing Microsoft Excel Software, using the whole data set, which includes: all days post-implant (Day5, Day6, Day7, Day10, and Day14) and all three mice lineages (Swiss, BALB/c, and C57BL/6). Based on the global median cut off, the results of each implant were categorized as “low” (below the global median) or “high” (above the global median). The categorical data were then used to calculate the proportion (percentage) of implants with biomarker levels above the global median cut-off. The curve comprising the ascendant frequencies of biomarkers was created for each day after implant and referred to as biomarkers signatures. The biomarkers with frequencies above the 75th percentile were considered relevant and highlighted by bold underline format. Microsoft Excel Software was used to assemble radar charts and final graphical art.

#### Biomarker Network Analyses and Circus Plot Assembling

The biomarker network was disclosure based on significant correlations indices identified for pairs of cell subset attributes (CD45^+^, Ly^+^, F4/80^+^, CD11c^+^, CD3–CD49^+^, CD3^+^, CD3^+^CD4^+^, and CD3^+^D8^+^). Those data were referred to as Source or Target biomarkers (we have named the “x” parameters as a “source” attribute and the “y” parameters as a “target” attribute) by the Pearson’s correlation test. The GraphPad Prism software (version 5.03, San Diego, CA, United States) was used to calculate correlations indices (*p* and *r*) further employed to construct a two-dimensional matrix of source and target biomarkers for each mouse lineage along the days post-implant. The significant correlations at *p* < 0.05) were highlighted according to the “*r*” score (positive correlations, *r* > 0, plain background, or negative correlations, *r* < 0, dashed background). Microsoft Excel Software was used to assemble all correlation matrices. Biomarker networks were further assembled using circus plot design to underscore the associations involving source biomarkers with increased levels (proportion above the global median cut-off >75%, thick lines) apart from those biomarkers without (proportion below the global median cut-off <75%, thin lines). The circus plot approach was used to illustrate the correlation between the biomarkers in each mice lineage (Swiss, BALB/c, and C57BL/6) during the follow-up (Day 5, Day 6, Day 7, Day 10, and Day 14). The open source software Cytoscape (version 3.1.1) was used to assemble the connecting edges between pairs of biomarkers, according to Shannon et al. (2003). Each biomarker was tagged as source (left side – counterclockwise) or target (right side – clockwise) around a circular layout. The networks were customized in a circus plot format using Microsoft PowerPoint Software.

## Results

### Timeline Changes on Histologic and Morphometric Features of Implanted Sponges

**FIGURE 2:**
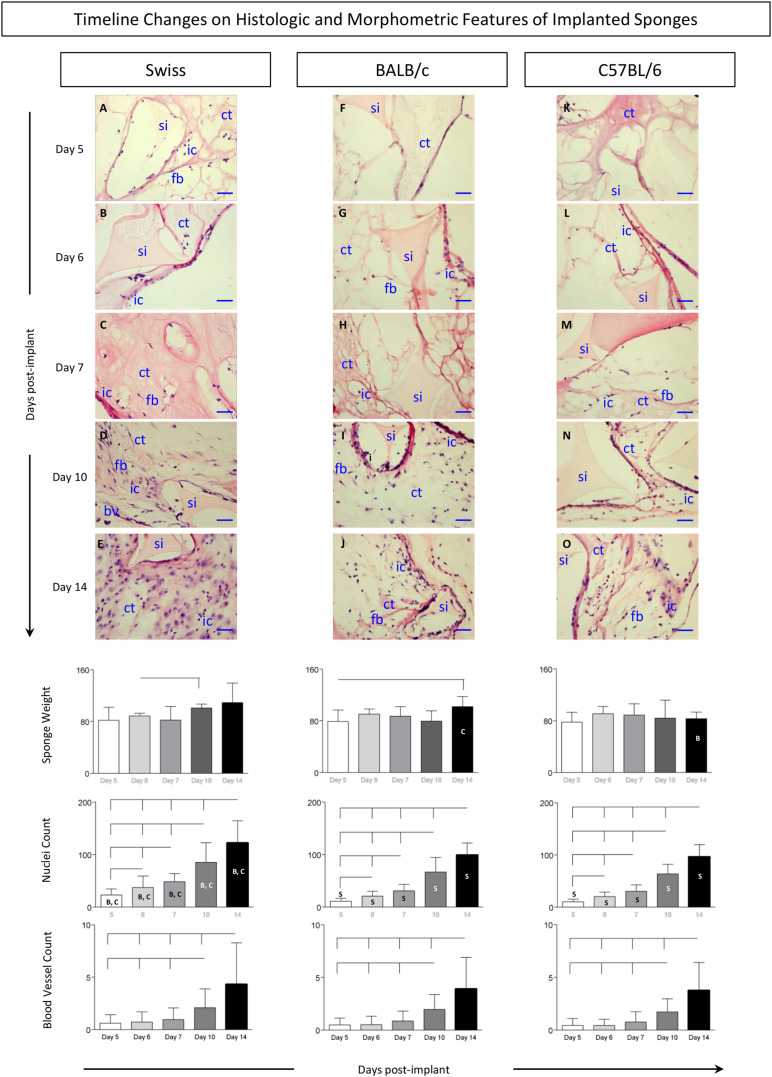
Histologic and morphometric features of implanted sponges. The sponge disks were subcutaneously implanted and removed at Day5, Day6, Day7, Day10, and Day14 post-implant. **(Top panels)** Sections of sponge implants were stained by the Hematoxylin/Eosin (HE) method, as described in Materials and Methods. Early and late events representing the most relevant changes in sponge implants removed from Swiss **(A–E)**, BALB/c **(F–J)**, and C57BL/6 mice **(K–O)** illustrate the progressive filling of sponge implants (si) with connective tissue (ct), inflammatory cells (ic), fibroblasts (fb), and blood vessels (bv) upon image acquisition under light microscopy (40X objective,— = 100 μm). **(Bottom panels)** Sponge weight, quantification of inflammatory infiltrate (cell nuclei), and blood vessel counts were monitored throughout the experimental procedures. Data are presented as mean ± standard deviation. Significant differences at *p* < 0.05 are underscored by connecting lines within the lineage and by letters “S,” “B,” and “C” for comparisons with Swiss, BALB/c, or C57BL/6 mice, respectively.

The kinetics of histological changes of sponge implants is shown in Figure 2. Descriptive imaging analysis demonstrated that, regardless the mice lineage, sponge implant displayed gradual increase of fibrovascular stroma from Day5 to Day14. Detailed analysis of the time courses revealed lineage-specific differences in the histological and morphometric profiles. In general, no changes in sponge weight were observed throughout the experimental follow-up (Figure 2, bottom panel). Conversely, the fibrovascular tissue presented early kinetics in Swiss (Figures 2A–E) as compared to BALB/c (Figures 2F–J) and C57BL/6 mice (Figures 2K–O), progressively populating the sponge implant (si) with connective tissue (ct), inflammatory cells (ic), fibroblasts (fb), and blood vessels (bv). As early as Day5, the generation of a connective capsule could be observed around the sponge implant (Figure 1). Up to Day7, the inner compartment of the sponge matrices was not completely filled and low numbers of inflammatory cells could be identified (Figures 2C,H,M). From Day10 on, higher inflammatory infiltrate could be identified in Swiss mice (Figures 2D,E) as compared to BALB/c (Figures 2I,J) and C57BL/6 mice (Figures 2N,O). Statistical analyses of nuclei count further support these findings (Figure 2, bottom panels). Angiogenesis was more evident at late stages of sponge implantation with no differences between lineages (Figure 2, bottom panels).

### Kinetics of Infiltrating Leukocytes Harvested From Sponge Implants

**FIGURE 3:**
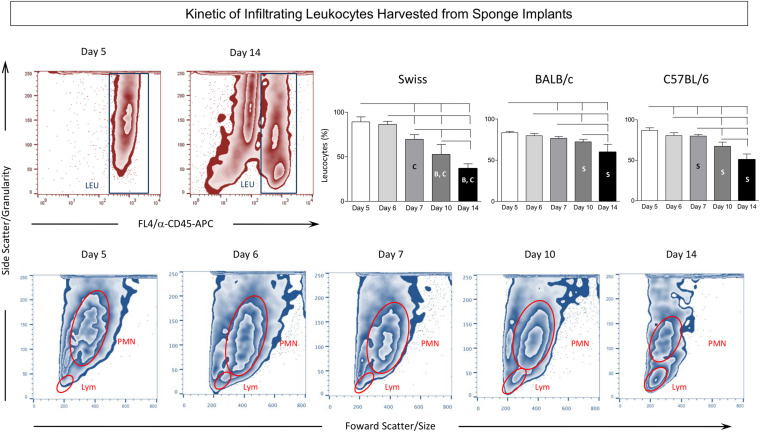
Kinetics of infiltrating leukocytes harvested from sponge implants. The sponge disks were subcutaneously implanted and removed at Day5, Day6, Day7, Day10, and Day14 post-implant. They were gently squeezed and cell suspension stained with anti-CD45 monoclonal antibody to selective flow cytometry quantification of leukocytes among the cells harvested from sponge implants. **(Top panels)** Representative zebra plots illustrate the decreasing predominance of leukocytes (LEU) among the cells harvested from sponge implants in Swiss mice. Statistical analysis further supports this finding and demonstrates that this phenomenon was more prominent in Swiss mice. Data are presented as mean ± standard deviation. Significant differences at *p* < 0.05 are underscored by connecting lines within the lineage and by letters “S”, “B”, and “C” for comparisons with Swiss, BALB/c, or C57BL/6, respectively. **(Bottom panels)** Follow-up analysis of flow cytometry morphometric features (Size – Forward Scatter/FSC and Granularity – Side Scatter/SSC) illustrated the progressive change in the leukocyte inflammatory infiltrate from general polymorphonuclear leukocytes (PMN) at Day5 toward a classical lymphocytic profile (LYM) at Day14.

Figure 3 shows the timeline changes on LEU infiltration removed from sponge implants. Data analysis demonstrated a progressive decrease in the frequency of LEU from Day5 to Day14 after implantation (Figure 3, top panels). Intergroup comparison demonstrated that Swiss presented the lower values at late time-points (Day10 and Day14) as compared to BALB/c and C57BL/6 mice. Representative Flow Cytometry zebra plots illustrate this phenomenon. Although the decrease on LEU infiltration was observed in all lineages, it was more pronounced in Swiss mice as compared to BALB/c and C57BL/6 mice. Moreover, detailed analysis revealed a progressive shift of the LEU infiltration from general polymorphonuclear leukocytes (PMN) at Day5 toward a classical lymphocytic profile (Lym) at Day14 (Figure 3, bottom panels).

### Dynamics of Leukocyte Subset Changes Harvested From Sponge Implants

**FIGURE 4:**
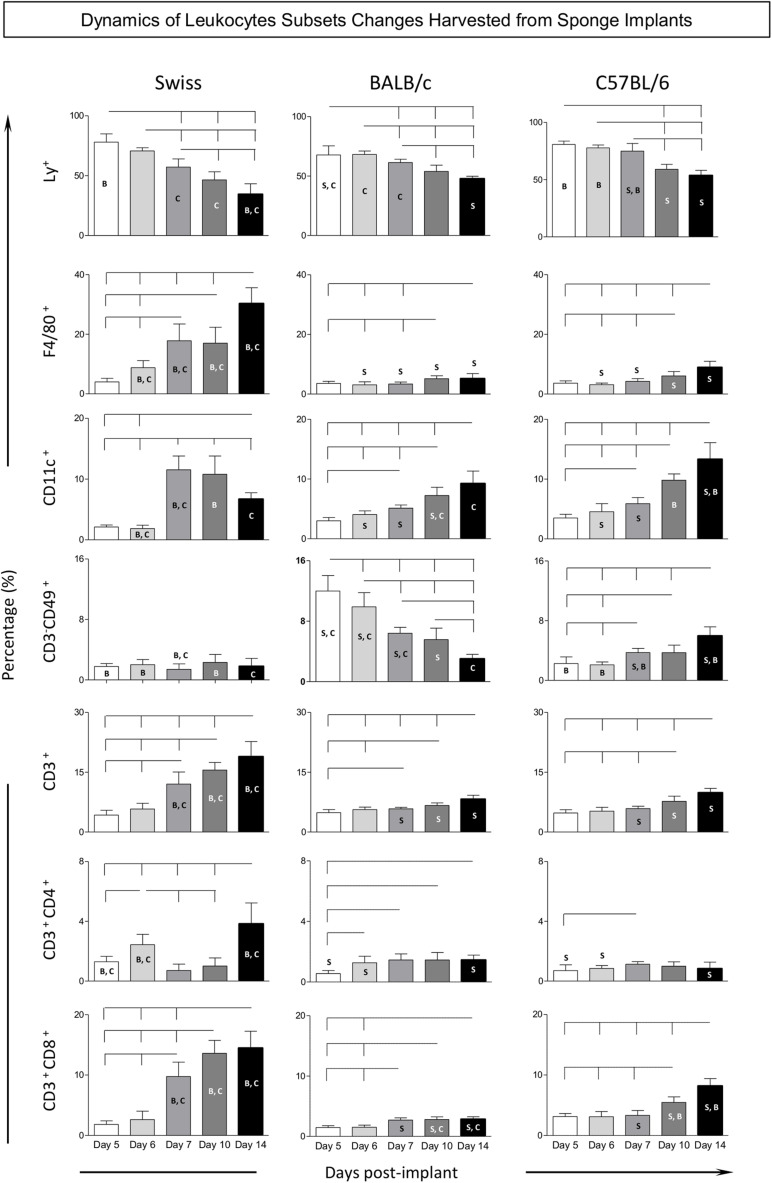
Kinetics of leukocyte subsets harvested from sponge implants. The sponge disks were subcutaneously implanted and removed at Day5, Day6, Day7, Day10, and Day14 post-implant. They were gently squeezed and cell suspension stained with monoclonal antibody for selective flow cytometry quantification of leukocytes subsets (CD45^+^): innate immunity cells [neutrophils (LY^+^), macrophages (F4/80^+^), dendritic cells (CD11c^+^), and NK-cells (CD3^–^ CD49^+^)], along with the adaptive immunity cells [T-cells (CD3^+^), CD4^+^T-cells (CD3^+^CD4^+^), and CD8^+^T-cells (CD3^+^CD8^+^)]. Data are reported as mean percentage of each cell subset ± standard deviation along the days post-implant. Intragroup and intergroup comparisons were assessed. Significant differences at *p* < 0.05 are underscored by connecting lines within the lineage and by letters “S,” “B,” and “C” for comparisons with Swiss, BALB/c, or C57BL/6 mice, respectively.

Flow cytometry immunophenotyping assay was performed to further characterize the kinetics of distinct LEU subsets harvested from the sponge implants. For this purpose, the frequency of innate immunity cells [neutrophils (Ly^+^), macrophages (F4/80^+^), dendritic cells (CD11c^+^), and NK-cells (CD3^–^CD49^+^)], along with the adaptive immunity cells [T-cells (CD3^+^), CD4^+^T-cells (CD3^+^CD4^+^), and CD8^+^T-cells (CD3^+^CD8^+^)], were quantified among the LEU (CD45^+^) harvested from the sponge implants (Figure 4).

Data analysis demonstrated a gradual decrease of neutrophils (Ly^+^), over time. In fact, there was a significant drop in Swiss mice among almost all time points. In BALB/c and C57BL/6 mice, the decrease of neutrophils (Ly^+^) was generally observed when comparing the late time-points (Day10 and Day14) to early time-points (Day5, Day6, and Day7). Intergroup comparison demonstrated that Swiss mice presented lower frequency of neutrophils (Ly^+^) at late time-points (Day10 and Day14) as compared to BALB/c and C57BL/6 mice (Figure 4).

Analysis of macrophages (F4/80^+^) in Swiss mice revealed a prominent significant increase over time among almost all time-points evaluated. In BALB/c and C57BL/6 mice, the increase of macrophages (F4/80^+^) was usually observed at late time-points (Day10 and Day14) as compared to early time-points (Day5, Day6, and Day7). Intergroup comparison demonstrated that, generally, starting at Day6, Swiss mice presented higher frequency of macrophages (F4/80^+^) as compared to BALB/c and C57BL/6 mice (Figure 4).

The frequency of dendritic cells (CD11c^+^) in Swiss mice revealed a significant increase at Day7 with subsequent decrease toward Day14. In BALB/c and C57BL/6 mice, the analysis of dendritic cells (CD11c^+^) showed progressive increase over time toward Day14. Intergroup comparison demonstrated that C57BL/6 mice presented a higher frequency of dendritic cells (CD11c^+^) at late time-points (Day14) as compared to Swiss and BALB/c mice (Figure 4).

No significant changes in the frequency of NK-cells (CD3^–^CD49b^+^) were observed in Swiss mice, which remained at low values throughout the experimental follow-up. A slight increase of NK-cells (CD3^–^CD49b^+^) was observed in C57BL/6 mice during the experimental evaluation toward Day14. Conversely, BALB/c mice displayed an early increase of NK-cells (CD3^–^CD49b^+^) at Day5 with progressive decrease toward Day14. Intergroup comparison demonstrated that BALB/c mice presented the highest frequency of NK-cells (CD3^–^CD49b^+^) at early time-points (Day5, Day6, and Day7) as compared to Swiss and C57BL/6 mice. However, the C57BL/6 mice displayed the highest frequency of NK-cells (CD3^–^CD49b^+^) at late time-point (Day14) as compared to Swiss and BALB/c mice (Figure 4).

A progressive increase in the frequency of T-cells (CD3^+^) was observed in Swiss mice over time among all time-points evaluated. Slighter changes were observed in BALB/c and C57BL/6 mice, with increased percentage of T-cells (CD3^+^) identified particularly at late time-points (Day10 and Day14) as compared to early time-points (Day5, Day6, and Day7). Intergroup comparison demonstrated that, starting at Day7, Swiss mice presented higher frequency of T-cells (CD3^+^) as compared to BALB/c and C57BL/6 mice (Figure 4).

Generally, the kinetics of CD4^+^T-cells (CD3^+^CD4^+^) and CD8^+^T-cells (CD3^+^CD8^+^) resemble the profile observed for T-cells (CD3^+^), with some particularities. Slighter changes were observed for CD4^+^T-cells (CD3^+^CD4^+^) in C57BL/6 mice, while a lower frequency of CD8^+^T-cells (CD3^+^CD8^+^) was observed in BALB/c mice (Figure 4).

### Changes in the Cytokine Microenvironment in the Sponge Implants

**FIGURE 5:**
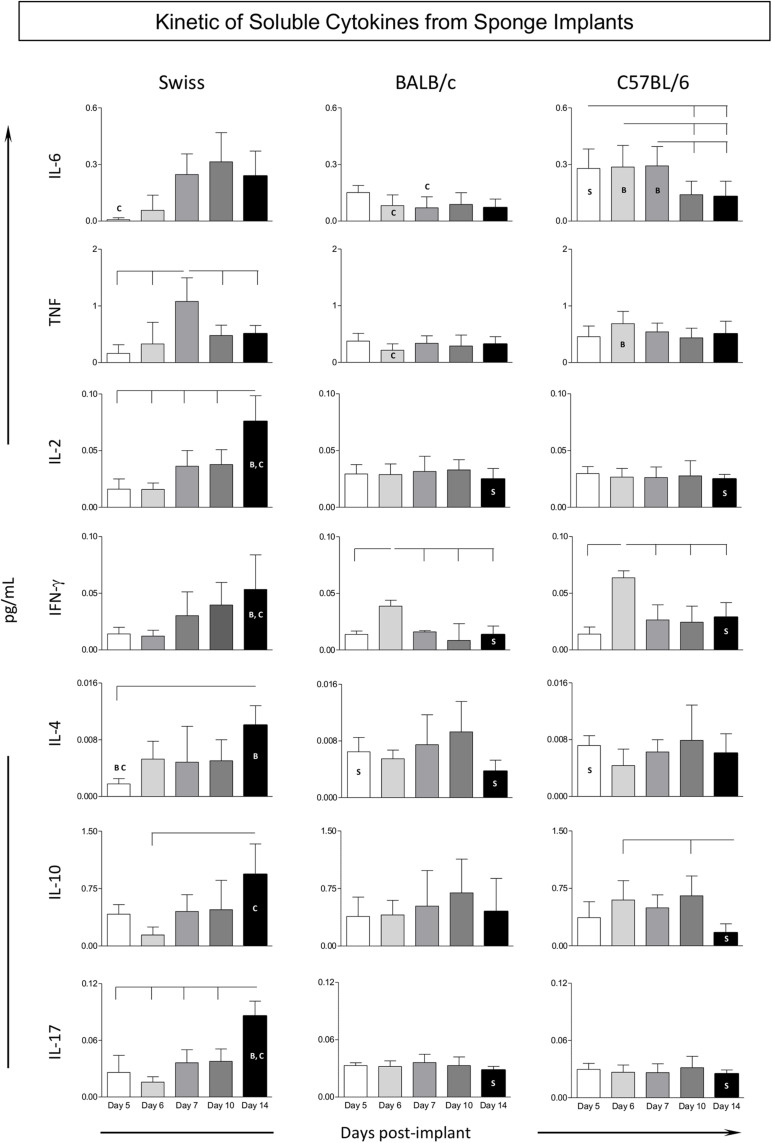
Kinetics of soluble cytokines from sponge implants. The sponge disks were subcutaneously implanted and removed at Day5, Day6, Day7, Day10, and Day14 post-implant. They were homogenized and soluble cytokine levels were measured by Cytometric Bead Array. Data are reported as mean levels (pg/mL) ± standard deviation along the days post-implant. Intragroup and intergroup comparisons were assessed. Significant differences at *p* < 0.05 are underscored by connecting lines within the lineage and by letters “S,” “B,” and “C” for comparisons with Swiss, BALB/c, or C57BL/6 mice, respectively.

The kinetics of the cytokine profile observed in the sponge implants is shown in Figure 5. Data analysis demonstrated that, in general, Swiss mice displayed a prominent late cytokine production, from Day7 (TNF) on, reaching higher levels at Day 14, with mixed pattern, including pro-inflammatory and regulatory mediators (IL-2/IL-4/IL-10/IL-17). Conversely, BALB/c and C57BL/6 mice mounted a more restricted cytokine response with minor changes along the day post-implants, despite the discrete production of cytokine observed in BALB/c mice, higher levels of IFN-γ at Day6, with subsequent decrease along with up-regulation of IL-4 toward late stages of sponge implants. C57BL/6 mice showed up-regulated levels of pro-inflammatory cytokines (IL-6/IFN-γ) at early stages. Intergroup comparisons pointed out that Swiss mice presented higher mixed cytokine late profile at Day14 as compared to BALB/c and C57BL/6 mice (Figure 5), whereas BALB/c and C57BL/6 mice presented a more prominent cytokine response at early stages of sponge implants (Figure 5).

### Kinetics of Ascendant Biomarker Signature in the Sponge Implants

**FIGURE 6:**
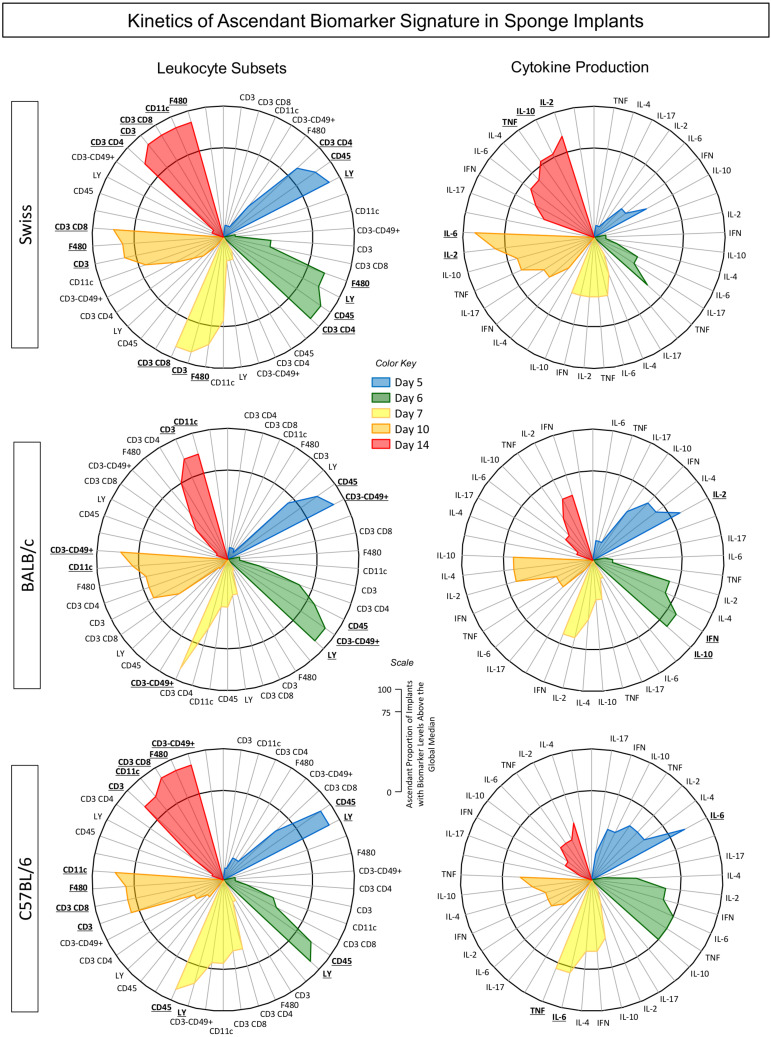
Kinetics of ascendant biomarker signature in sponge implants. The sponge disks were subcutaneously implanted and removed at Day5, Day6, Day7, Day10, and Day14 post-implant. They were gently squeezed and cell suspension stained for selective quantification of leukocytes subsets by Flow Cytometry. In parallel experiments, the sponge implants were homogenized for measurement of cytokine production by Cytometric Bead Array. Data are reported as biomarker signature according to Luiza-Silva et al. (2011). The biomarker signatures were determined using the frequency of biomarker level above the global median cut-off defined for each biomarker. The data of biomarkers displayed in the Figure show the frequency of those presenting high levels (above the global median cut-off). Radar charts were used to compile and summarize the ascendant proportion of implants with biomarker levels above the global median for each cell subset (CD45^+^, Ly^+^, F4/80^+^, CD11c^+^, CD3–CD49^+^, CD3^+^, CD3^+^CD4^+^, and CD3^+^CD8^+^) as well as each soluble cytokine (IL-6, TNF, IL-2, IFN-γ, IL-4, IL-10, and IL-17), at different time-points post-implant [Day5 (blue), Day6 (green), Day7 (yellow), Day10 (orange), and Day14 (red)] for Swiss, BALB/c, and C57BL/6 mice. The biomarkers with frequencies above the 75th percentile were considered relevant and highlighted in bold underline format.

Aimed at characterizing the overall profile of changes in phenotypic and functional features of the immune response in the sponge throughout the days post-implant, the ascendant biomarker signatures (from the lowest to the highest frequency) were assembled as shown in Figure 6. Data analysis corroborated most findings detected by conventional statistical approaches. The kinetics of ascendant biomarker signature in sponge implants comprises two sets of data referred to as LEU subsets and cytokine production analysis.

The biomarker signatures of LEU subsets revealed that Swiss mice presented the most prominent immune response to the sponge implant with a higher number of cell surface biomarkers with increased levels from Day5 on and recruitment of cytotoxic immune response starting at Day7. Conversely, BALB/c and C57BL/6 mice presented lower number of cell surface biomarkers with increased levels. While BALB/c mice showed early activation of the innate response with a low number of cell surface biomarkers, mediated by neutrophils (Ly^+^) and NK-cells (CD3^–^CD49^+^), C57BL/6 mice also presented a low number of cell surface biomarkers, but with a predominant neutrophilic recruitment (Figure 6).

The analysis of cytokine production demonstrated that Swiss mice exhibited a more prominent immune response mediated by higher levels of cytokine at late time-points (Day10 and Day14), mediated by a mixed profile (IL-2, IL-6, TNF, and IL-10). Contrariwise, BALB/c and C57BL/6 mice showed a more restricted pattern of cytokine response, confined at early time-points, mediated by IL-2/IFN-γ/IL-10, and IL-6/TNF, respectively.

A snapshot of these data allowed identifying the time-points after the sponge implant with a lower number of altered biomarkers (cell surface markers and cytokines). In fact, for Swiss mice, the Day5/Day7 and Day5/Day6/Day7 showed the lowest number of altered cell surface biomarkers and cytokines, respectively. For BALB/c mice, the Day7 and Day7/Day10/Day14 were accompanied with the lowest number of altered cell surface biomarkers and cytokines, respectively. The analysis of C57BL/6 mice pointed out Day5/Day6 and Day6/Day10/Day14 as the time-points with lower numbers of altered cell surface biomarkers and cytokines, respectively.

### Connectivity Framework Among Phenotypic and Functional Biomarkers in Sponge Implants

Systems biology approaches have become useful tools to explore large data sets to provide a more comprehensive overview of interactions among distinct cell subsets and soluble factors. To better determine the degree of connectivity among cell surface biomarkers and cytokines at distinct time-points post sponge implants, a correlation matrix was constructed to compile the significant association between pairs of “source” and “target” attributes and underscore the positive and negative connection edges. These data can be taken together with the biomarker signature profile to assemble a comprehensive biomarker circus scenario.

**FIGURE 7:**
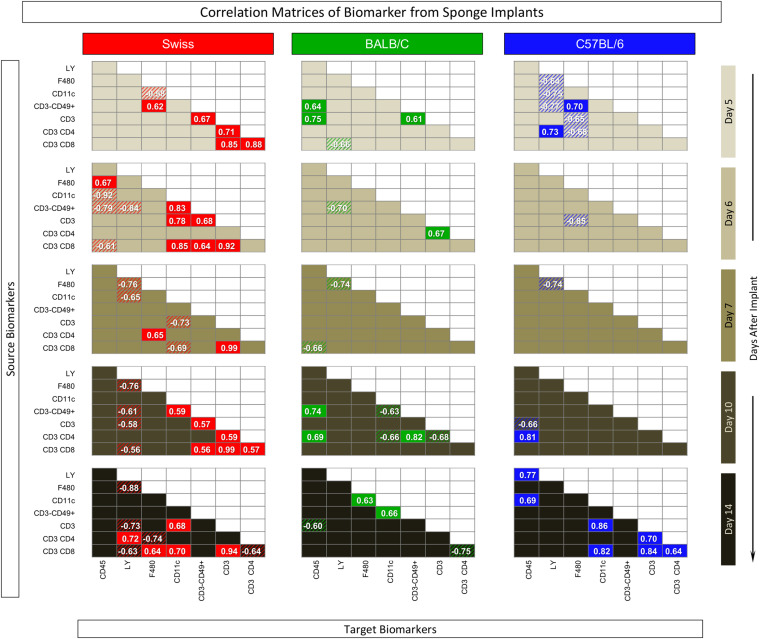
Correlation matrix of biomarker from sponge implants. The sponge disks were subcutaneously implanted and removed at Day5, Day6, Day7, Day10, and Day14 post-implant. Selective quantification of leukocyte subsets and measurement of cytokine production were assessed by flow cytometry. Pearson’s correlation test was applied to identify significant time-point-dependent connectivity between pairs of biomarkers (“source” and “targets”). Correlation indices (*p* and *r*), along with the 95% confidence, were used to identify significant associations (*p* < 0.05) and highlight “negative” (*r* < 0, dashed background) and “positive” (*r* > 0, plain background) connections apart from non-significant correlations (empty background).

**FIGURE 8:**
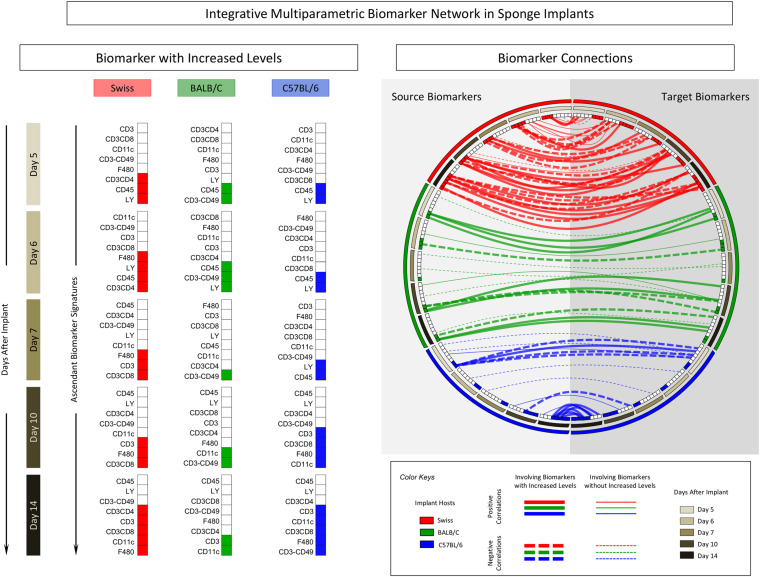
Integrative multiparametric biomarker network in sponge implants. The sponge disks were subcutaneously implanted and removed at Day5, Day6, Day7, Day10, and Day14 post-implant. Following the selective quantification of leukocyte subsets and measurement of cytokine production by flow cytometry, the ascendant biomarker signature was built to identify biomarkers with increased levels and the Pearson’s correlation test used to identify the significant associations between pairs of attributes (“source” and “target” biomarkers). Multicolor diagrams underscore the biomarkers with increased levels along the time-points post-implants. Circus plots assemble the integrative multiparametric biomarker network, pointing out the “negative” (dashed lines) and “positive” (continuous lines) connections involving biomarkers without (thin lines) or with (thick lines) increased levels.

The correlation keyboard illustrating the correlation matrix is provided in Figure 7 and an integrative multiparametric biomarker network presented in Figure 8.

The correlation matrix analysis demonstrated that Swiss mice presented, apart from Day7, a large number of positive and negative correlations, evenly distributed along the time-post implant. BALB/c mice presented lower connectivity between pairs of biomarkers that, apart from Day7, were also evenly distributed along the time-post implant. In C57BL/6 mice, the biomarker connectivity was particularly confined at the Day5 and Day14 poles (Figure 7).

The panoramic analysis of biomarker connectivity scenario by integrative multiparametric circus plot further confirms these findings, demonstrating that the connectivity involving biomarkers with increased levels was much more evident and evenly distributed along distinct time-points post-implants in Swiss mice as compared to BALB/c and C57BL/6 mice, which show lower connectivity profiles. Moreover, while BALB/c presented similar distribution along the time-points post-implants, C57BL/6 mice displayed a polar distribution of connectivity, mainly focused at Day5 and Day14 (Figure 8).

Together with the biomarker signature profiles, the biomarker network structure can provide a rational approach to support the selection of the ideal time-point and mouse lineage conforming to the specific experimental purposes. A proposal of a rational strategy to select the ideal time-point for each mouse lineage may comprise major criteria to avoid bias that could lead to tendentious or skewed results. Three major criteria could be considered during the selection of time-points for each mouse lineage, including: (i) time-points with a low number of phenotypic biomarkers with increased levels and no recruitment of cytotoxic immune response; (ii) time-points with minor cytokine production, and (iii) time-points with a lower number of connections involving biomarkers with increased levels. Using such criteria, our findings indicated that Day5 for Swiss, Day7 for BALB/c, and Day6 for C57BL/6 mice seem to be the best time-points to be used in future investigations to support the rational choice of the best model for biomolecule screening.

## Discussion

The sponge implant model is a feasible strategy for screening bioactive molecules. Although the sponge model has been extensively studied over the last few decades (Andrade et al., 1987, 1997; Bailey, 1988; Barcelos et al., 2004, 2009; Belo et al., 2004; Mendes et al., 2007, 2009; Xavier et al., 2010; Agrawal et al., 2011; Marques et al., 2011; Moura et al., 2011a,b,c; Saraswati et al., 2011; Pereira et al., 2012, 2014; Alhaider et al., 2014a, b; Almeida et al., 2014; Cassini-Vieira et al., 2014, 2016; Guedes-da-Silva et al., 2015; Michel et al., 2015), little is known about the kinetics of phenotypic and functional profile of the immune response triggered by the sponge implants in distinct mouse lineages. The present study aimed to provide a detailed characterization of phenotypic and functional changes in the sponge implant microenvironment that could ultimately support the selection of the best mouse lineage and time-point for screening assays.

The kinetics of histological changes of sponge implants showed that, regardless of the mice lineage, the fibrovascular tissue progressively occupied the pores of the sponge matrix, filling the implant trabeculae with inflammatory cells, fibroblasts, connective tissue, and blood vessels. Neovascularization, the presence of endothelial cells and fibroblasts, as well as the formation of connective tissue were identified along the days post-implants. Our results showed that, in all mice lineages, a connective capsule could be observed around the sponge implant and low numbers of inflammatory cells could be identified at early time-points (Day5, Day6, and Day7), while angiogenesis was more evident at late time-points (Day10 and Day14). Some lineage-specific differences were observed, as were the early fibrovascular tissue neoformation and a late inflammatory response in Swiss mice as compared to BALB/c and C57BL/6 mice. Differences among the number of blood vessels and vascular area in sponge implants removed from Swiss mice have been previously reported by Marques et al. (2011).

The dynamics of inflammation in the sponge implant microenvironment was first characterized based on the analysis of LEU subsets harvested from the sponge implants. Data revealed that a clear reduction in the percentage of LEU over time was observed, regardless of the mouse lineage. Moreover, at early time-points, the inflammatory infiltrate was mainly composed of polymorphonuclear cells with a progressive shift toward mononuclear cells at late time-points. Similar results were observed for Swiss mice by Moura et al. (2011a) and Guedes-da-Silva et al. (2015) when we compared data at Day4, Day7, and Day14. Interestingly, Swiss mice presented the highest percentage of LEU at early time-points and also the lowest frequency of LEU at late time-points when compared to the inbred strains.

Our study has described the kinetics of distinct LEU subsets harvested from the sponge implants. The findings showed a decreased frequency of neutrophils (Ly^+^) and an increased percentage of macrophages (F4/80^+^) and T-cells (CD3^+^) at late time-points in all three lineages, consistent with what occurs in the inflammatory processes (LeBert and Huttenlocher, 2014). Remarkably, Swiss mice showed the most prominent decrease in neutrophils (Ly^+^) over time and the highest increase in macrophages (F4/80^+^). Several studies have used the levels of the lysosomal enzyme NAG as a biomarker to infer macrophage recruitment or activation in sponge implants (Andrade et al., 1987; Bailey, 1988; Xavier et al., 2010; Marques et al., 2011; Moura et al., 2011a,b; Pereira et al., 2012; Almeida et al., 2014; Cassini-Vieira et al., 2014, 2016; Guedes-da-Silva et al., 2015; Michel et al., 2015). Using this approach, the findings of Marques et al. (2011) corroborate our results, demonstrating higher NAG activity in implants from Swiss as compared to BALB/c and C57BL/6 mice.

Overall, while the Swiss mice exhibited a more prominent immune response leading to high levels cytokines at late time-points, BALB/c and C57BL/6 mice showed a distinct pattern of immune response to the sponge implants. The panoramic analysis of biomarker connectivity corroborated these findings and should be taken together with the biomarker signature profile as they demonstrate that the connectivity involving biomarkers with increased levels was much more evident and evenly distributed along distinct time-points post implants in Swiss mice. While BALB/c mice showed an early activation of the innate response, mediated by neutrophils (Ly^+^) and NK-cells (CD3^–^CD49^+^) with a controlled cytokine profile and presented similar connectivity distribution along the time-points post-implants, C57BL/6 mice presented a typical early pro-inflammatory response with persistent neutrophilic involvement and displayed a polar distribution of connectivity, mainly focused at Day5 and Day14.

Based on the kinetics of phenotypic and functional changes observed in each mouse lineage, a rational approach can be established to select the ideal time-point for each mouse lineage. Three major criteria could be considered during the selection of time-points, including: (i) time-points with a lower number of phenotypic biomarkers with increased levels and no recruitment of cytotoxic immune response, (ii) time-points with minor cytokine production, and (iii) time-points with a lower number of connections involving biomarkers with increased levels. These parameters are also reinforced by results described in biomarker signature analysis and network analysis, which indicated a useful approach to identifying time points when these criteria are met. Since the sponge implant can induce a natural inflammatory reaction at the implant site, these criteria could guide the appropriate experimental model choice (low inflammatory events). Therefore, minor inflammatory events presented at the sponge implant provide an appropriate microenvironment for testing biomolecules in this model by reducing the expected interference regarding the inflammation triggered by the sponge implant. Using these criteria, Day5 post-implant for Swiss mice, Day7 for BALB/c mice, and Day6 for C57BL/6 mice seem to be the best times to be applied in future investigations to support the rational choice of the best model for biomolecule screening.

Taken together, the details and changes in features, the kinetics of the overall profile of biomarkers elicited on sponge implants, the characterization of phenotypic and functional aspects of the sponge implant in different mouse strains is essential and relevant since it has become increasingly recognized that genetic background will result in profoundly different phenotypes even when the same stimulus is given (Marques et al., 2011). Since different mouse lineages influence various physiological and pathological parameters, it is important to study these differences to establish criteria for the applications of the model, whether for testing anti-inflammatory drugs or biomolecule screening.

In conclusion, the sponge model elicited the formation of an inflammatory response that differed among distinct mice lineages. These differences demonstrated that genetic background strongly influences the inflammatory process temporally, quantitatively, and qualitatively. The data presented here describe the kinetics of changes in the phenotypic and functional features in the sponge model, using efficient methodologies that have never before been employed to this purpose, which also has a great potential for use in biomolecule screening. Our findings provided evidence to support the rational choice of the best model for biomolecule screening. This information is relevant for guiding and supporting the selection of the ideal time-point for each mouse lineage conforming to the specific experimental proposal. Taking into account the particular immunological microenvironment at distinct time-points of the sponge model, it would be possible to optimize the protocols for specific screening purposes, such as identification of therapeutic biomolecules, selection of antigens and adjuvants, and follow up on the innate and adaptive immune responses to vaccine candidates.

## Data Availability Statement

All datasets generated for this study are included in the article/Supplementary Material.

## Ethics Statement

The studies involving animals were reviewed and approved by Ethical Committee for Animal Studies from Federal University of Ouro Preto by protocol number 014/2011.

## Author Contributions

ML, LR, RA-S, MM, LM, and DS-L performed the experiments. ML, LR, RA-S, MM, LM, OM, RM, JL, and PS wrote the manuscript. ML, RC-O, WD, AR, OM-F, SM, DS-L, and RG reviewed the manuscript. ML, RG, and DS-L drafted and critically evaluated the manuscript. All authors contributed to the article and approved the submitted version.

## Conflict of Interest

The authors declare that the research was conducted in the absence of any commercial or financial relationships that could be construed as a potential conflict of interest.

## Supplementary Material

Supplementary Figure 1 Immunophenotypic analysis of CD45^+^ leukocytes by analyzing the side scatter parameter (SSC) versus FL4 (CD45 +) **(A)** in sponge implants to identify: TCD3^+^CD4^+^ lymphocytes in a FL1 x FL3 **(B)** graph; TCD3^+^CD8^+^ lymphocytes using a FL4 x FL3 **(C)** graph; NK cells through the selection of CD3^–^ lymphocytes in a FL2 x FL3 **(D)** chart, followed by the labeling of CD49b^+^ cells in a FL2 x FL1 **(E)** chart; dendritic cells in a FL2 x FL3 graph; macrophages F4/80^+^ in a FL3 x FL1 **(G)** graph; and neutrophils by analyzing the side scatter parameter (SSC) versus FL4 (Ly^+ High^) **(H)**.
