# Corrigendum: Kinetics of Phenotypic and Functional Changes in Mouse Models of Sponge Implants: Rational Selection to Optimize Protocols for Specific Biomolecules Screening Purposes

**DOI:** 10.3389/fbioe.2021.660117

**Published:** 2021-03-03

**Authors:** Mariana Ferreira Lanna, Lucilene Aparecida Resende, Rodrigo Dian de Oliveira Aguiar-Soares, Marina Barcelos de Miranda, Ludmila Zanandreis de Mendonça, Otoni Alves de Oliveira Melo Júnior, Reysla Maria da Silveira Mariano, Jaqueline Costa Leite, Patricia Silveira, Rodrigo Corrêa-Oliveira, Walderez Ornelas Dutra, Alexandre Barbosa Reis, Olindo Assis Martins-Filho, Sandra Aparecida Lima de Moura, Denise Silveira-Lemos, Rodolfo Cordeiro Giunchetti

**Affiliations:** ^1^Laboratório de Biologia das Interações Celulares, Departamento de Morfologia, Universidade Federal de Minas Gerais, Belo Horizonte, Brazil; ^2^Laboratório de Pesquisas Clínicas, Programa de Pós-Graduação de Ciências Farmacêuticas, Universidade Federal de Ouro Preto, Ouro Preto, Brazil; ^3^Laboratório de Biomateriais e Patologia Experimental, Instituto de Ciências Exatas e Biológicas, Universidade Federal de Ouro Preto, Ouro Preto, Brazil; ^4^Grupo de Pesquisa em Imunologia Celular e Molecular, Instituto de Pesquisa René Rachou, Fundação Oswaldo Cruz, Belo Horizonte, Brazil; ^5^Grupo Integrado de Pesquisas em Biomarcadores, Instituto de Pesquisa René Rachou, Fundação Oswaldo Cruz, Belo Horizonte, Brazil; ^6^Departamento de Medicina, Universidade José Rosário Vellano, Belo Horizonte, Brazil

**Keywords:** sponge implant model, biomolecules screening, dynamics of phenotypic and functional features, immunophenotyping, cytokines

In the original article, there was an error. We incorrectly referenced the sponge as being associated to “Vitafoam Ltd., Manchester, United Kingdom.” The correct reference is “Rei das Espumas, Belo Horizonte, Brazil.”

A correction has been made to the **Materials and Methods**, subsection **Sponge Implants** paragraph one:

Disk-shaped (4 mm × 8 mm) polyether-polyurethane sponges (Rei das Espumas, Belo Horizonte, Brazil) were soaked overnight in 70% v/v ethanol and boiled in distilled water for 15 min prior to implantation. Mice were anesthetized by intra-peritoneal injection of ketamine (150 mg kg^−1^) plus xylazine (10 mg kg^−1^) and the dorsal fur shaved and the skin wiped with 70% v/v ethanol. The sponge disks were subcutaneously implanted throughout a 1-cm long dorsal mid-line incision and the animals were monitored daily for discomfort/distress or any signs of opportunistic infection. Sponge implants were removed for histological/morphometric analysis, flow cytometry, immunophenotyping, and soluble cytokine measurements at Day5, Day6, Day7, Day10, and Day14 after implantation. The compendium of the experimental design, study groups, timeline, and illustrated images of sponge implants are provided in Figure 1.

The authors apologize for this error and state that this does not change the scientific conclusions of the article in any way. The original article has been updated.

