# Supplementary figures and images for: Kinetics of Phenotypic and Functional Changes in Mouse Models of Sponge Implants: Rational Selection to Optimize Protocols for Specific Biomolecules Screening Purposes
Source: Front Bioeng Biotechnol. 2020 Dec 2;8:538203. doi: 10.3389/fbioe.2020.538203 (PMC7738572; doi:10.3389/fbioe.2020.538203)

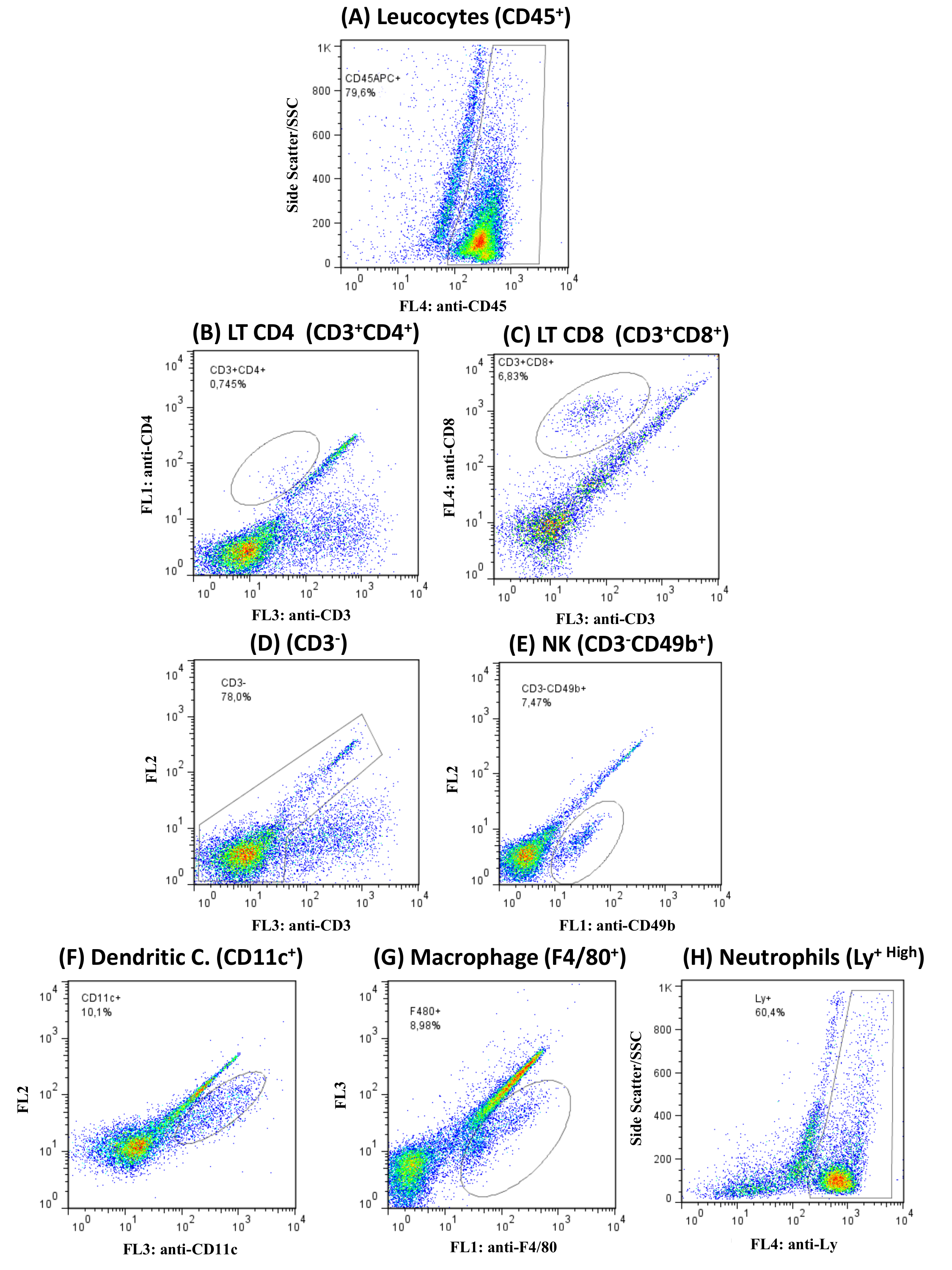

Supplement: Supplementary Figure 1 — Immunophenotypic analysis of CD45+ leukocytes by analyzing the side scatter parameter (SSC) versus FL4 (CD45 +) (A) in sponge implants to identify: TCD3+CD4+ lymphocytes in a FL1 x FL3 (B) graph; TCD3+CD8+ lymphocytes using a FL4 x FL3 (C) graph; NK cells through the selection of CD3– lymphocytes in a FL2 x FL3 (D) chart, followed by the labeling of CD49b+ cells in a FL2 x FL1 (E) chart; dendritic cells in a FL2 x FL3 graph; macrophages F4/80+ in a FL3 x FL1 (G) graph; and neutrophils by analyzing the side scatter parameter (SSC) versus FL4 (Ly+ High) (H). [file Image_1.TIF]
